# Left Atrium Wall-mapping Application for Wall Thickness Visualisation

**DOI:** 10.1038/s41598-018-22089-z

**Published:** 2018-03-08

**Authors:** Jing-Yi Sun, Chun-Ho Yun, Greta S. P. Mok, Yi-Hwa Liu, Chung-Lieh Hung, Tung-Hsin Wu, Mohamad Amer Alaiti, Brendan L. Eck, Anas Fares, Hiram G. Bezerra

**Affiliations:** 1 Department of Biomedical Imaging and Radiological Sciences, National Yang Ming University, Taipei, Taiwan; 2 Department of Medicine, Mackay Medical College, and Mackay Medicine Nursing and Management College, Taipei, Taiwan; 3 Department of Radiology, Mackay Memorial Hospital, Taipei, Taiwan; 4 Biomedical Imaging Laboratory, Department of Electrical and Computer Engineering, Faculty of Science and Technology, University of Macau, Macau, SAR China; 5 Department of Internal Medicine (Cardiology), Yale University, New Haven, CT USA; 6 Department of Internal Medicine (Cardiology), Mackay Memorial Hospital, Taipei, Taiwan; 7 Institute of Clinical Medicine, and Cardiovascular Research Center, Taipei, Taiwan; 8 Cardiovascular Department, University Hospitals Case Medical Center, Cleveland, OH USA

## Abstract

The measurement method for the LA wall thickness (WT) using cardiac computed tomography (CT) is observer dependent and cannot provide a rapid and comprehensive visualisation of the global LA WT. We aim to develop a LA wall-mapping application to display the global LA WT on a coplanar plane. The accuracy, intra-observer, and inter-observer reproducibility of the application were validated using digital/physical phantoms, and CT images of eight patients. This application on CT-based LA WT measures were further validated by testing six pig cardiac specimens. To evaluate its accuracy, the expanded maps of the physical phantom and pig LA were generated from the CT images and compared with the expanded map of the digital phantom and LA wall of pig heart. No significant differences (*p* > 0.05) were found between physical phantom and digital phantom as well as pig heart specimen and CT images using our application. Moreover, the analysis was based on the LA physical phantom or images of clinical patients; the results consistently demonstrated high intra-observer reproducibility (ICC > 0.9) and inter-observer reproducibility (ICC > 0.8) and showed good correlation between measures of pig heart specimen and CT data (*r* = 0.96, *p* < 0.001). The application can *p*rocess and analyse the LA architecture for further visualisation and quantification.

## Introduction

Abnormal electrical signals can interfere with the electrical conducting system of the heart and are among the major factors causing atrial fibrillation (AF). Recent studies show that the chronic remodeling of the left atrium (LA) architecture under AF—such as the atrial wall thickness, chamber volume, cross-section diameter, and shape of the ostium of pulmonary vein (PV)—may lead to maintenance of multiple electrical re-entrant wavelets and further provide a firm substrate for AF in the LA^1–4^. If no proper treatment is given to cease the progression of the remodeling process, it would not only accelerate the deterioration of atrial function, but also lead to other chronic complications such as heart failure and stroke^5,6^.

The use of cardiac rhythm control medication with catheter ablation is one of the effective ways to treat AF, especially in the early phase^7–10^. In addition to the experience of the physician, the treatment outcome of AF by catheter ablation is closely correlated with the LA electrical-signal distribution and LA architecture^11–13^. Therefore, diagnostic tools, including cardiac catheterisation, electrocardiography (ECG), electrophysiological study (EPS), magnetic resonance imaging (MRI) and cardiac computed tomography (CT), are needed during the treatment process. Among these tools, cardiac CT is the most popular application for LA surface rendering to provide coarse correlation and localisation for a three-dimensional (3D) EPS map.

Cardiac CT has high spatial and temporal resolutions and can provide detailed images of the LA. Therefore, researchers have used cardiac CT to detect LA-related architectural structures^7,14–17^, including LA chamber volume and characteristics of the PVs^18–21^, without detailed information about the global LA wall thickness, which is a major concern of catheter ablation. The inhomogeneity of the atrium wall thickness is also a reason for the retention of abnormal electrical signals^22,23^.

In previous studies, most methods of LA wall assessment on cardiac CT images were primarily for a single point of the LA wall^7,15,16^. The wall thickness was determined according to the linear distance between particular chosen points of interest on the images. In addition to the disadvantages of being time consuming and prone to the influence of the subjective view of the observer, these methods could not provide rapid and comprehensive visualisation of the LA wall thickness^14,17^. Thus, the aim of the present study was to develop an LA wall-mapping application that could realise global wall thickness visualisation and quantification on a coplanar plane. We demonstrated its accuracy, intra-observer, and inter-observer reproducibility on studies of LA phantoms and clinical cardiac CT images.

## Methods

### Study design

**Figure 1:**
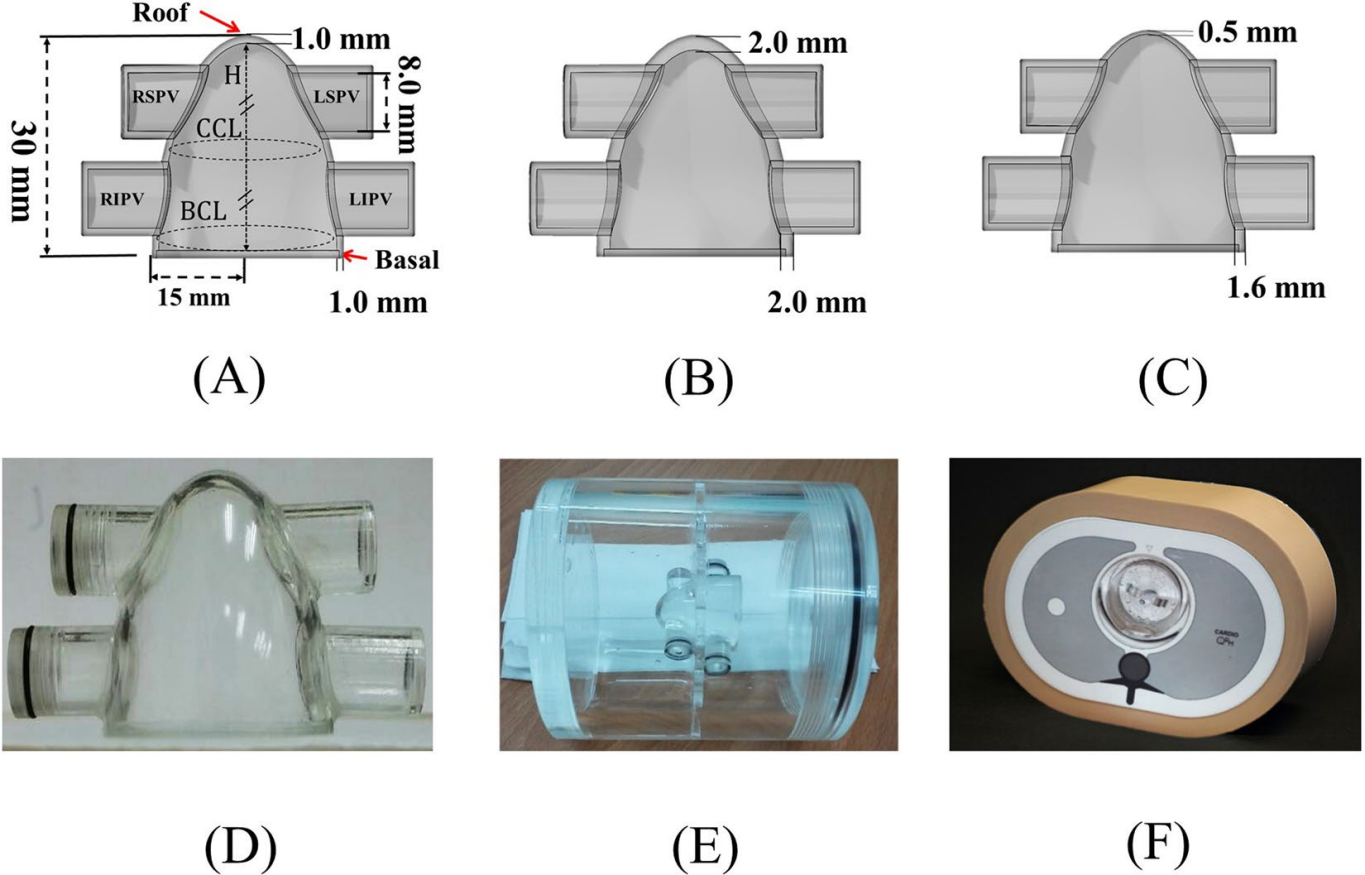
Sectional views of the LA digital phantoms with (**A**) a homogeneous wall thickness of 1.0 mm from the roof to the base, (**B**) a homogeneous wall thickness of 2.0 mm, (**C**) an inhomogeneous gradient wall thickness with a wall thickness of 0.5 mm at the roof and a gradual increase to 1.6 mm at the base. (**D**) Sectional view of the physical acryl phantom corresponding to LA digital phantom (**A**). (**E**) LA physical phantom fixed in a cylindrical water phantom with a 10-cm diameter. (**F**) Cylindrical water phantom placed in the QRM cardio phantom. H: height; CCL: central circumference length; BCL: basal circumference length; LSPV: left superior pulmonary vein; LIPV: left inferior pulmonary vein; RSPV: right superior pulmonary vein; RIPV: right inferior pulmonary vein.

We first designed a LA digital phantom using a 3D modelling software. As shown in Fig. 1, the LA digital phantom is modelled based on the average LA architecture of an adult (height = 30 mm, basal diameter = 30 mm). The top is the roof, and the bottom is the basal area where the LA and mitral valve (MV) are connected. The four tubes, two on the left side and two on the right side of the phantom, simulated four PVs connected to the LA with an inner diameter of 8 mm. The LA wall thickness was designed to be distributed inhomogeneously, ranging from 0.5 mm to 2mm^24^. We also designed three LA digital phantoms with different characteristic wall thicknesses: a homogenous wall thickness phantom with a wall thickness of 1.0 mm, another homogenous wall thickness phantom with a wall thickness of 2.0 mm, and an inhomogeneous gradient wall thickness phantom whose thickness was 0.5 mm at the LA roof and gradually increased to 1.6 mm at the base. These three phantoms can be directly expanded through image-space transformation, and the global LA wall thickness data can be displayed on a coplanar plane. In this study, the expanded map of the LA digital phantom served as the gold standard, i.e., LA_gold standard_.

We have made physical acryl phantoms, i.e. LA physical phantoms, based on the aforementioned three LA digital phantoms, using a computer numerical control (CNC) milling machine. The CNC technology allowed us to control the errors between the LA digital phantom and the LA physical phantom to be within 0.02 mm. To simulate the X-ray attenuation by the surrounding tissues in the thorax under CT scans, we first fixed the LA physical phantom in a cylindrical water phantom with a diameter of 10 cm and then put the cylindrical water phantom in a quantitative risk management (QRM) cardio phantom. The QRM cardio phantom was composed of human tissue-equivalent materials, including spine, lung, chest bone, and soft tissue. Its attenuation coefficients are similar to those of human tissues^25^.

To further validate the accuracy of the LA wall-mapping application, CT scans of the LA physical phantom were performed. An expanded wall thickness map, i.e. LA_measurement_, was generated for the CT images by the LA wall-mapping application. Then, the calculated wall volume, chamber volume, central circumference length (CCL), basal circumference length (BCL) and areas of the ostium of four PVs of the LA physical phantom were compared with the LA_gold standard_. Each set of the LA wall map was repeatedly analysed five times. The mean and standard error were calculated, and an independent two-sample *t* test was performed to evaluate the difference between the LA_measurement_ and the LA_gold standard_. A p-value less than 0.05 was considered to indicate statistical significance.

**Figure 2:**
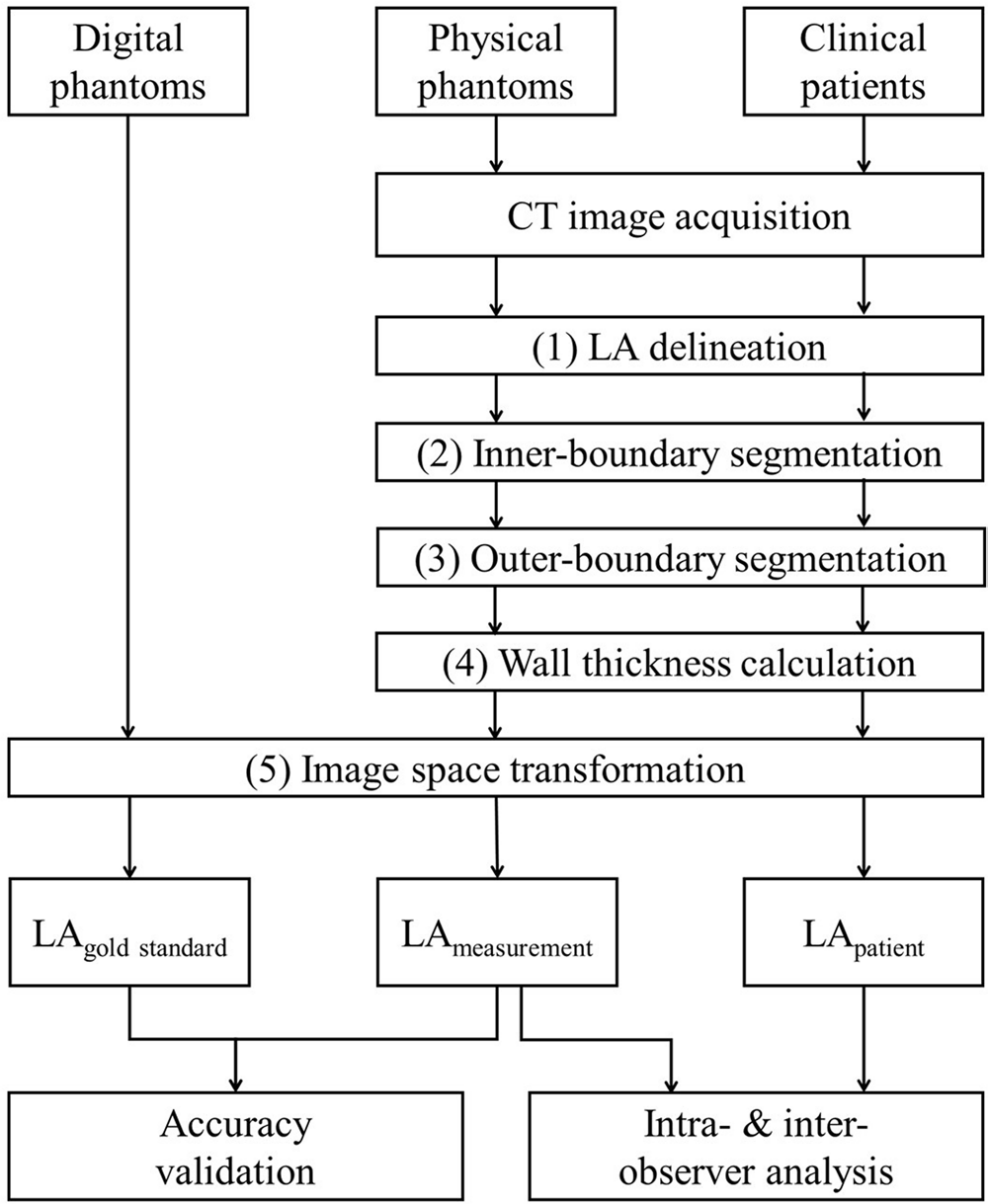
The study design flowchart.

In addition, cardiac CT images of eight patients were retrospectively collected to evaluate the clinical feasibility of the method. The CT images with metal artifacts and high quantum noise were excluded in this study. The intra-observer and inter-observer reproducibility of the LA wall-mapping application were evaluated on three LA physical phantoms and on the eight patients. A statistical analysis of the intra-class correlation coefficient (ICC) was performed with 95% confidence intervals. The intra-observer reproducibility was measured by a repeatability evaluation based on two analyses conducted by the same operator 1 month apart. The inter-observer reproducibility was measured by a reproducibility assessment based on the analysis of the same images by two different observers. The study design flowchart is shown in Fig. 2.

### Cardiac CT images

We used a dual-source CT system (Definition, Siemens Medical Systems, Forchheim, Germany) for the experiments. For the phantom study, the scan protocol included retrospective ECG-gating, tube voltage of 120kVp, 320 reference mA with automatic exposure control, pitch of 0.28 and 2 × 128 × 0.6 mm collimation. The raw data were reconstructed with a slice thickness of 0.75 mm, increment of 0.4 mm, and a common cardiac kernel (B26f) associated with the field-of-view of 160 mm, a 512 × 512 matrix, and a resolution of 0.31 mm/pixel. The ECG monitor demo mode function was applied during CT scan of the phantoms with a preset heartbeat of 60 bpm. The scan parameters for clinical patients were similar, with the pitch automatically adjusted from 0.28 to 0.38 according to the patient heartbeat. The scans range of *z*-axis was from the level of 1 cm below the carina to the dome of the diaphragm. To determine the peak enhancement in the ascending aorta, a pre-scan test bolus study was performed by injection of 10 mL of nonionic iodinated contrast material (Iopamiro, Bracco Industria Chimicas. p.a., of Milano, Italy) followed by 20 mL of normal saline, using a power injector at a rate of 5 mL/s. Then, the remaining 50 mL of nonionic iodinated contrast material was injected using a power injector at a rate of 5 mL/s. Scans were started with a delay equal to the time to peak enhancement plus 8 seconds. After the cardiac CT images were acquired, images obtained at the LA diastolic phase, i.e. the ventricular systolic phase, which is between 30% and 40% of the R-R interval phases, were reconstructed.

In addition, we assessedthe ability and validity of our mapping application on LA wall thickness measurements by using porcine model. The study protocol in accordance with relevant guidelines and regulations was approved by Case Western reserve University’s Institutional Animal Care and Use Committee (IACUC) as previously described^26^. Six female Yorkshire pigs (40–50 kg, 13–15 weeks of age) were procured from Local vendors and were used in this study. The pig heart CT data were acquired using a spectral detector CT (Philips Healthcare, Cleveland, OH) with following scan parameters and contrast medium injection protocol: ECG-gated heart scan with kVp: 120, mAs: 400; bolus tracking technique was used by placing an ROI in the LV cavity and setting a 150 hounsfield unit (HU) trigger and taking the scan 10 s after this trigger. Total dose of contrast medium (Optiary 350) was varied from 30–60 ml mixed with 30 ml normal saline followed by 30 mL normal saline at a rate of 5 mL/sec through an 18- or 20-gauge catheter in the ear vein. The pig heart CT images were reconstructed at LA diastolic phase (30% of cardiac cycle) with 0.67 mm in slice thickness and overlap of 0.33 mm. Subsequent transmural LA wall thickness analysis and comparisons were made between our LA map application and pig heart specimens by defining regional point-by-point manner (4 points in each specimen).

### LA wall-mapping application

The LA wall-mapping application was programmed to be a set of automatic applications with the capability to process and analyse the global LA wall thickness and visualise the map on a coplanar plane. It also allows quantification of cardiac CT images. The application consists of five procedures.

Procedure 1 is called LA delineation and is the only procedure that needs manual input. Its purpose is to separate the PV and automatically set the isocentre of the LA chamber. Procedure 2 is inner-boundary segmentation, wherein the LA chamber containing contrast medium is automatically segmented using the region growing method through dilation from the isocentre. In combination with the Sobel method, it can delineate the inner-boundary of the LA^27^. By integrating the number of voxels within the inner-boundary, the LA chamber volume can be calculated. Procedure 3 is outer-boundary segmentation. An Otsu threshold-based algorithm performs outward dilation from the inner-boundary to look for the HU threshold between the LA wall and the surrounding soft tissue^14^. It then converges to the outer boundary between the LA wall and the surrounding soft tissue. Procedure 4 is to measure the wall thickness, which is the shortest distance between the inner and outer boundaries calculated using the Euclidean distance method^28^. The LA wall volume is calculated by integrating the number of voxels between the inner and outer boundaries. Procedure 5 is image-space transformation. Its purpose is to perform simultaneous localisation and visualisation of the LA wall thickness map and the 3D CT images on the same coplanar plane.

### Image-space transformation

**Figure 3:**
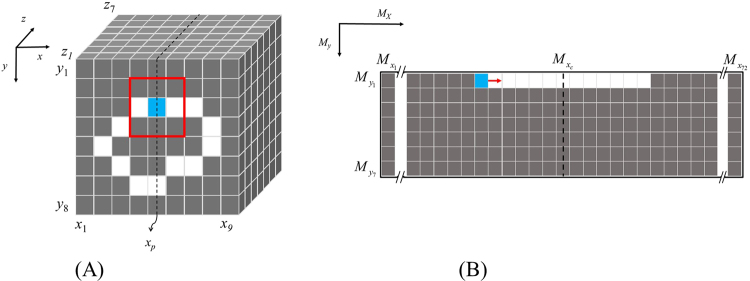
Schematic diagram of the image-space transformation. (**A**) The 3D image-space. White voxels belong to the LA wall, grey voxels belong to the background, and the blue voxel is the initial tracking point for the location of the LA wall thickness in the 3D image space. The red rectangleis the weighting matrix controlling the moving direction of the tracking point. (**B**) The 2D mapping space. The red arrow indicates the moving direction of mapping the LA wall thickness data onto the 2D mapping space in a certain slice.

To the best of our knowledge, an accurate and automatic image-space transformation algorithm of LA wall mapping for direct thickness visualisation has not yet been proposed in existing literature. After image pre-processing via procedures 1 to 4, image-space transformation (procedure 5) can be performed on the cardiac CT images. The purpose of procedure 5 is to convert the wall-thickness data obtained from 3D CT images to 2D expanded images. The schematic diagram of image-space transformation is shown in Fig. 3. First, an initial tracking point must be defined on the LA wall as the initial location (*x, y, z*_*a*_)_1_ (blue point on Fig. 3). This initial tracking point corresponds directly to the initial location (*M*_*x*_, *M*_*y*_)_1_ on the 2D mapping space. The initial tracking points are shown in equation 1:

1 $$\begin{array}{rcl}{M}_{x} & = & {M}_{{x}_{c}}-\sum _{i=1}^{{x}_{p}}\sum _{j=1}^{{y}_{8}}v({x}_{i},\,{y}_{j},{z}_{a})\\ {M}_{y} & = & {z}_{a},\end{array}$$

where *M*_*x*_and *M*_*y*_ are the coordinate values on the *x*-axis and *y*-axis, respectively, in the 2D mapping space; *M*_*x*c_ is the *x*-axis centre location in the 2D mapping space; *x*, *y*, *z* are the coordinate values on the *x*-axis, *y*-axis, and *z*-axis, respectively, in the 3D image space *v*; *x*_*p*_ is the *x*-axis location of the initial tracking point perpendicular to the *x*-axis in the 3D image space; and *a* is the slice for image-space transformation.

After the initial tracking point in each image space is defined, the next step is to map the LA wall-thickness data in the 3D image space to the 2D mapping space, pixel by pixel (equation 2).

2 $$M{({M}_{x},{M}_{y})}_{n}={v}_{d}{(x,y,{z}_{a})}_{n},$$

Here, *M* is the 2D mapping space, *v*_*d*_ represents the LA wall-thickness data in the 3D image space, and *n* is the number of the wall-thickness datapoint at slice *a*, initial *n* set by 1.

In the 2D mapping space, the tracking point moves to the right along the *M*_*x*_ axis. The motion of the tracking point in the 3D image space is defined by equation 3:

3 $$\begin{array}{rcl}v{(x,y,{z}_{a})}_{n+1} & = & {\rm{\max }}({v}_{b}[\begin{array}{ccc}(x-1,y-1,{z}_{a}) & (x,y-1,{z}_{a}) & (x+1,y-1,{z}_{a})\\ (x-1,y,{z}_{a}) & {(x,y,{z}_{a})}_{n} & (x+1,y,{z}_{a})\\ (x-1,y+1,{z}_{a}) & (x,y+1,{z}_{a}) & (x+1,y+1,{z}_{a})\end{array}]\\  &  & \times w[\begin{array}{ccc}7 & 8 & 1\\ 6 & 0 & 2\\ 5 & 4 & 3\end{array}]),\end{array}$$

where *v*_*b*_ is the binary matrix containing the tracking point and the eight neighbouring pixels surrounding it in the 3D image space, and *w* is the weight matrix that controls the moving direction of the tracking point. After the *v*_*b*_ × *w* matrix operation, the maximum value in the matrix is determined as the next tracking point location.

The first loop calculation is between equations 2 and 3 while *n* remains unchanged. Then, the tracking point moves to the next slice (*a* + 1) and the loop calculation backs to equation 1. The loop calculation continues until all the wall-thickness data on the 3D image spaceare transformed onto the 2D mapping space.

## Results

### Phantom studies

**Figure 4:**
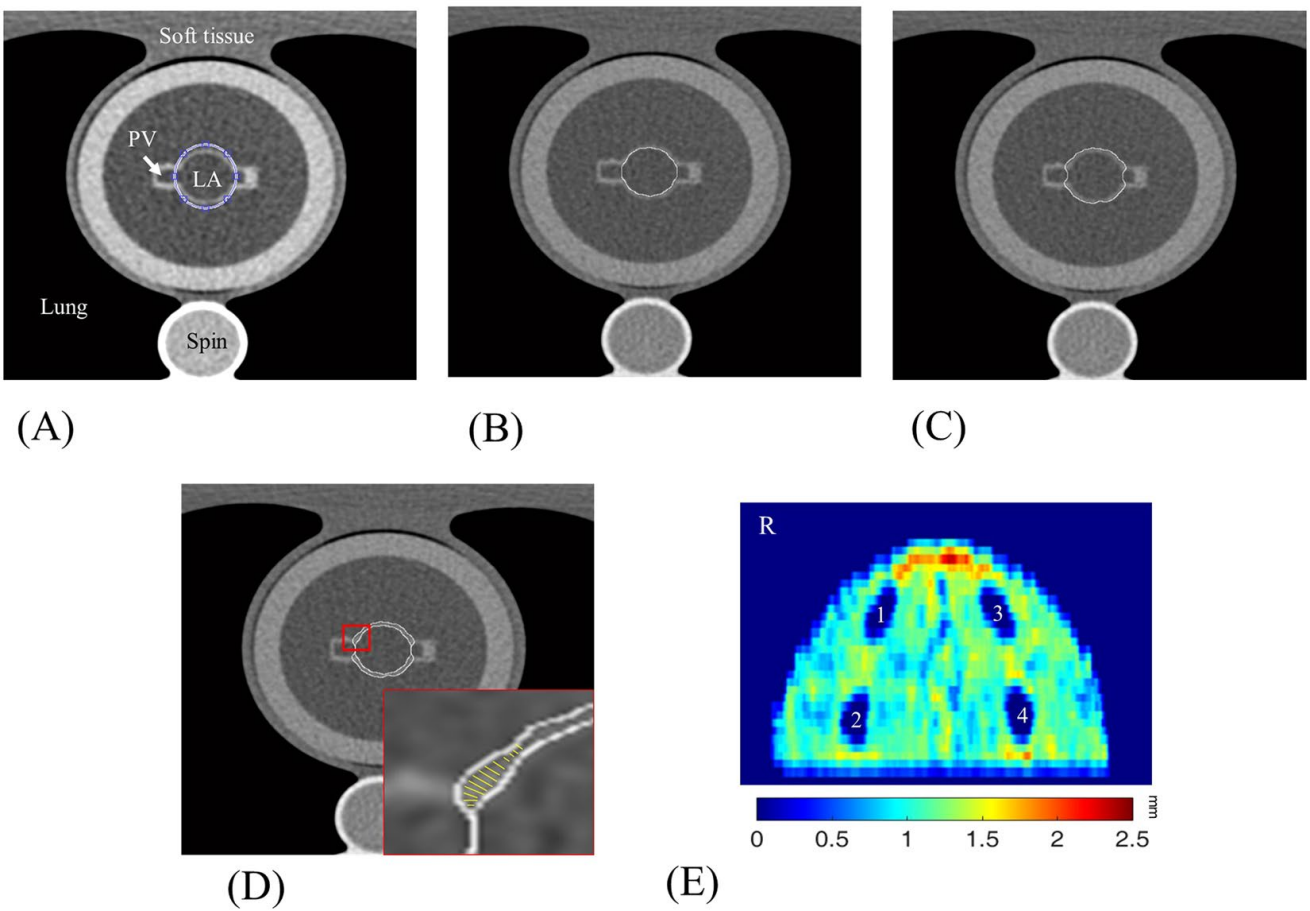
Procedures for processing and analysing the LA physical phantom using the LA wall-mapping application. (**A**) LA delineation; (**B**) inner-boundary segmentation; (**C**) outer-boundary segmentation; (**D**) wall thickness calculation; and (**E**) 2D wall map generation (four holes on the map are the pulmonary vein ostium). The colour bar shows the wall thickness.

**Figure 5:**
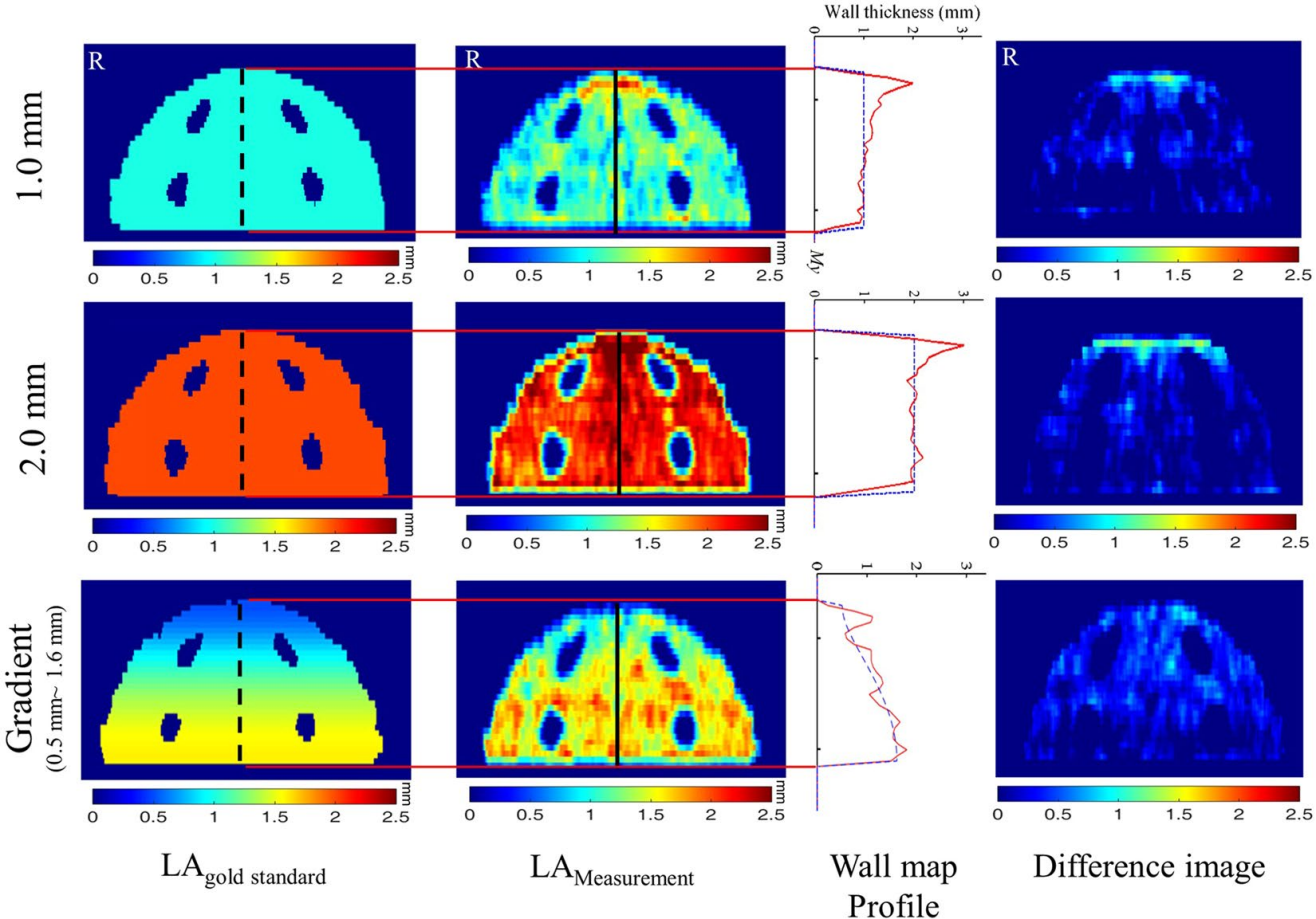
Comparison of the LA_gold standard_ (first column) with the LA_measurement_ (second column). The third column shows the corresponding wall-map profile. The blue dashed line is for LA_gold standard_ andthe red solid line for LA_measurement_. The last column showsthe difference map between the LA_gold standard_ and LA_measurement_. The results for the 1 mm, 2 mm, and gradient wall thicknessare shown in the top, middle, and bottom row, respectively. The colour bar shows thewall thickness.

**Figure 6:**
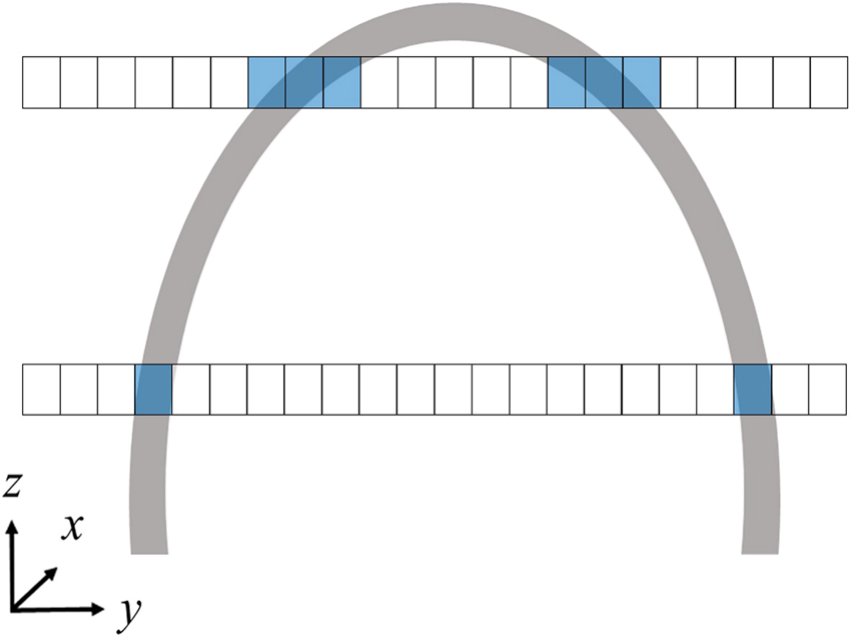
Partial volume effect on the LA wall. The horizontal bar indicates a CT slice, and the blue voxelsshow the LA wall thickness is overestimated, especially in the roof region.

In Fig. 4, we show the image analysis results for steps 1 to 5 of the LA physical phantom by sequence. Figure 5 shows the accuracy validation results of the expanded maps for the LA digital phantom and the CT images of the LA physical phantom. Comparing the subtracted map and corresponding profile, the distribution of the wall thickness from the CT measurement is similar to that of the gold standard, whereas the wall thickness at the roof appears to be overestimated. This overestimated is primarily attributed by the partial volume effect (PVE) in the CT images^29^ (Fig. 6). For the geometric comparison, the absolute error is obtained by subtracting the gold standard directly from the measurement, and the relative error is obtained by dividing the absolute error by the gold standard and multiplying by 100 (Table 1). A positive error indicates an overestimation, and a negative error indicates an underestimation. For all three LA phantoms, the measurement underestimated the chamber volume and height and overestimated the LA wall volume, CCL, BCL, and area of the four PVs. However, no statistically significant differences (*p* > 0.05) were observed. Table 2 shows the intra-observerand inter-observer reproducibility of the proposed method to analyse the related geometric indexes, demonstrating high intra-observer and inter-observer reproducibility (ICC > 0.9, 95% CI 0.80–0.99) for two independent measurements or two operators.

**Table 1:** Geometric comparison of the LA_gold standard_ and LA_measurement_.

	Wall thickness	LA_measurement_ (mean ± SD)	LA_gold standard_ (mean ± SD)	Absolute Error	Relative error (%)	*t* test (*p*)
Chamber volume (cm^3^)	1.0 mm	10.93 ± 0.47	11.10 ± 0.38	−0.17	−2%	0.13
	2.0 mm	8.94 ± 0.36	9.13 ± 0.38	−0.19	−2%	0.11
	Gradient	10.17 ± 0.40	10.32 ± 0.29	−0.15	−1%	0.18
Wall volume (cm^3^)	1.0 mm	2.73 ± 0.27	2.61 ± 0.16	0.12	5%	0.09
	2.0 mm	4.57 ± 0.20	4.42 ± 0.19	0.16	4%	0.05
	Gradient	3.53 ± 0.22	3.46 ± 0.18	0.07	2%	0.11
Height (mm)	1.0 mm	28.22 ± 0.59	28.94 ± 0.44	−0.72	−3%	0.06
	2.0 mm	26.41 ± 0.62	27.50 ± 0.59	−1.09	−4%	0.05
	Gradient	28.73 ± 0.41	29.29 ± 0.21	−0.56	−2%	0.06
CCL (mm)	1.0 mm	80.39 ± 0.90	80.06 ± 1.03	0.34	0.4%	0.58
	2.0 mm	72.06 ± 1.04	71.81 ± 0.94	0.26	0.4%	0.67
	Gradient	75.14 ± 1.00	74.73 ± 0.80	0.41	0.5%	0.34
BCL (mm)	1.0 mm	90.48 ± 1.11	90.29 ± 0.84	0.19	0.2%	0.55
	2.0 mm	87.35 ± 1.04	87.08 ± 0.71	0.27	0.3%	0.33
	Gradient	88.95 ± 1.05	88.81 ± 0.92	0.15	0.2%	0.57
RSPV (mm^2^)	1.0 mm	50.32 ± 4.30	48.25 ± 4.25	2.07	4%	0.47
	2.0 mm	54.56 ± 3.42	53.45 ± 1.91	1.11	2%	0.52
	Gradient	49.81 ± 3.50	47.89 ± 2.01	1.92	4%	0.31
RIPV (mm^2^)	1.0 mm	50.67 ± 1.22	49.46 ± 2.11	1.21	2%	0.30
	2.0 mm	54.44 ± 2.10	53.40 ± 3.42	1.04	2%	0.58
	Gradient	48.89 ± 2.27	47.96 ± 2.90	0.93	2%	0.53
LSPV (mm^2^)	1.0 mm	49.48 ± 2.43	48.13 ± 2.35	1.35	3%	0.41
	2.0 mm	54.72 ± 3.42	54.12 ± 3.60	0.62	1%	0.62
	Gradient	49.71 ± 3.75	48.32 ± 3.13	1.39	3%	0.67
LIPV (mm^2^)	1.0 mm	51.94 ± 2.09	50.88 ± 2.61	1.06	2%	0.49
	2.0 mm	53.53 ± 2.39	52.88 ± 3.45	0.65	1%	0.65
	Gradient	48.73 ± 3.45	47.21 ± 3.14	1.52	3%	0.63

CCL: central circumference length; BCL: basal circumference length. LSPV: left superior pulmonary vein; LIPV: left inferior pulmonary vein; RSPV: right superior pulmonary vein; RIPV: right inferior pulmonary vein.

**Table 2:** Intra-observer and inter-observer reproducibility of the LA wall-mapping application from the physical phantom study.

	Intra-observer ICC	95% CI	Inter-observer ICC	95% CI
Chamber volume	0.99	0.99–0.99	0.98	0.96–0.99
Wall volume	0.99	0.98–0.99	0.96	0.90–0.98
Wall thickness	0.97	0.89–0.99	0.94	0.87–0.97
Height	0.95	0.89–0.98	0.94	0.81–0.98
CCL	0.95	0.89–0.98	0.94	0.82–0.96
BCL	0.95	0.88–0.98	0.94	0.80–0.97
RSPV	0.94	0.83–0.96	0.92	0.81–0.95
RIPV	0.95	0.85–0.96	0.93	0.80–0.96
LSPV	0.94	0.83–0.96	0.93	0.80–0.96
LIPV	0.95	0.84–0.97	0.93	0.80–0.96

CCL: central circumference length; BCL: basal circumference length; LSPV: left superior pulmonary vein; LIPV: left inferior pulmonary vein; RSPV: right superior pulmonary vein; RIPV: right inferior pulmonary vein.

### Animals and clinical feasibility study

**Figure 7:**
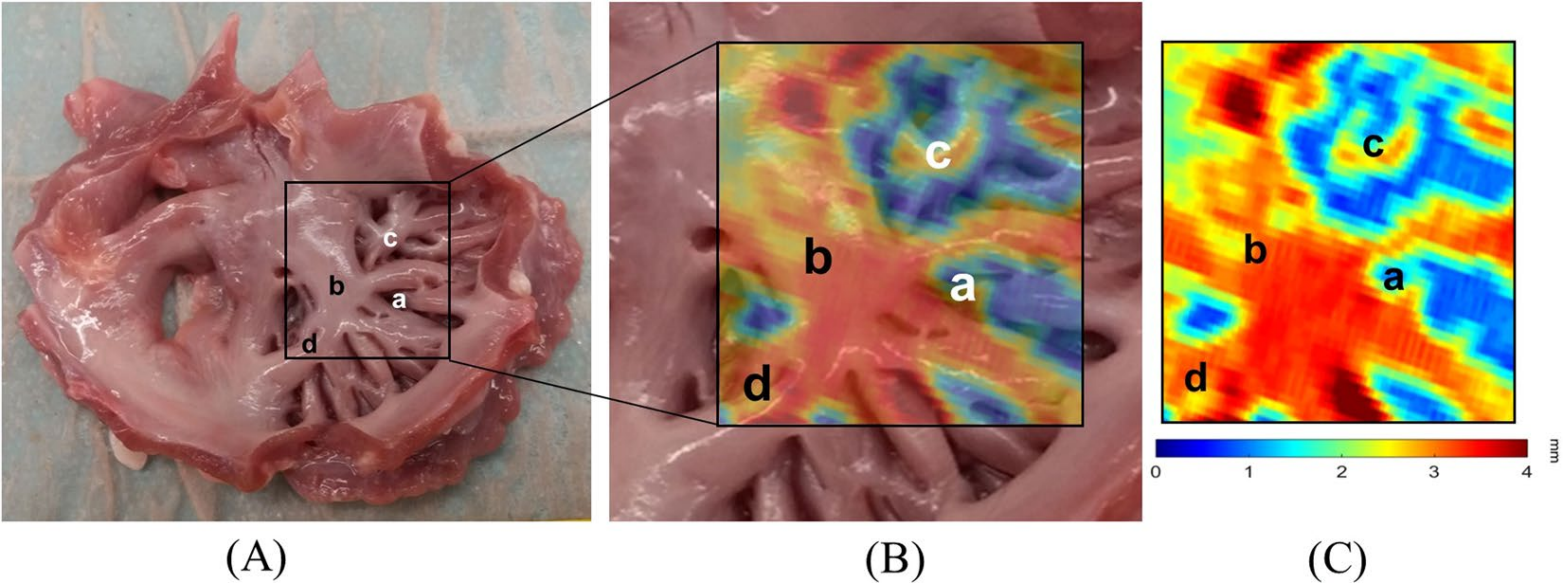
Comparison of (**A**) the pig heart specimen and (**B**) the fusion image with (**C**) the LA wall map. The LA wall thickness between specimen and CT image was (a) 0.31 mm vs. 0.51 mm, (b) 2.88 mm vs 2.76 mm, (c) 1.51 mm vs 1.53 mm, and (d) 3.13 mm vs 3.21 mm.

**Figure 8:**
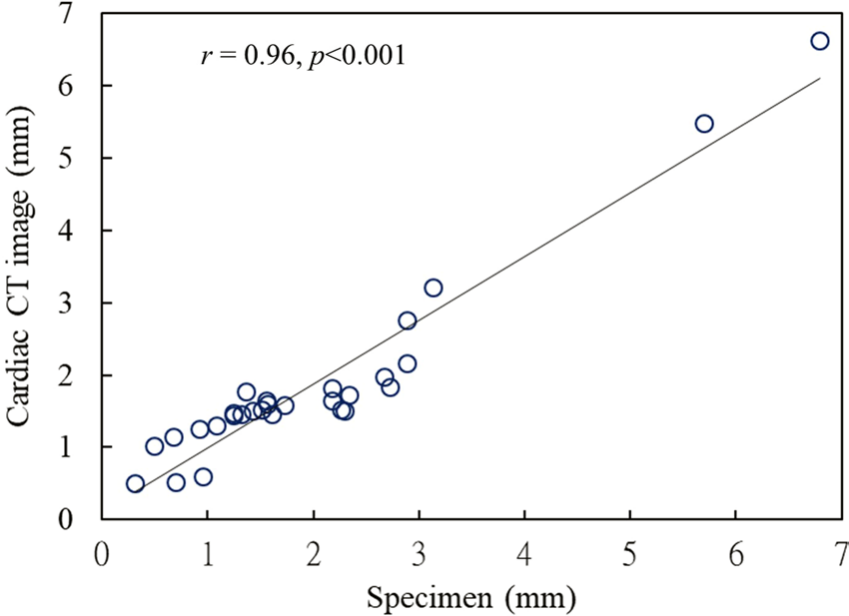
The correlation of LA wall thickness between the pig heart specimen and cardiac CT image.

**Figure 9:**
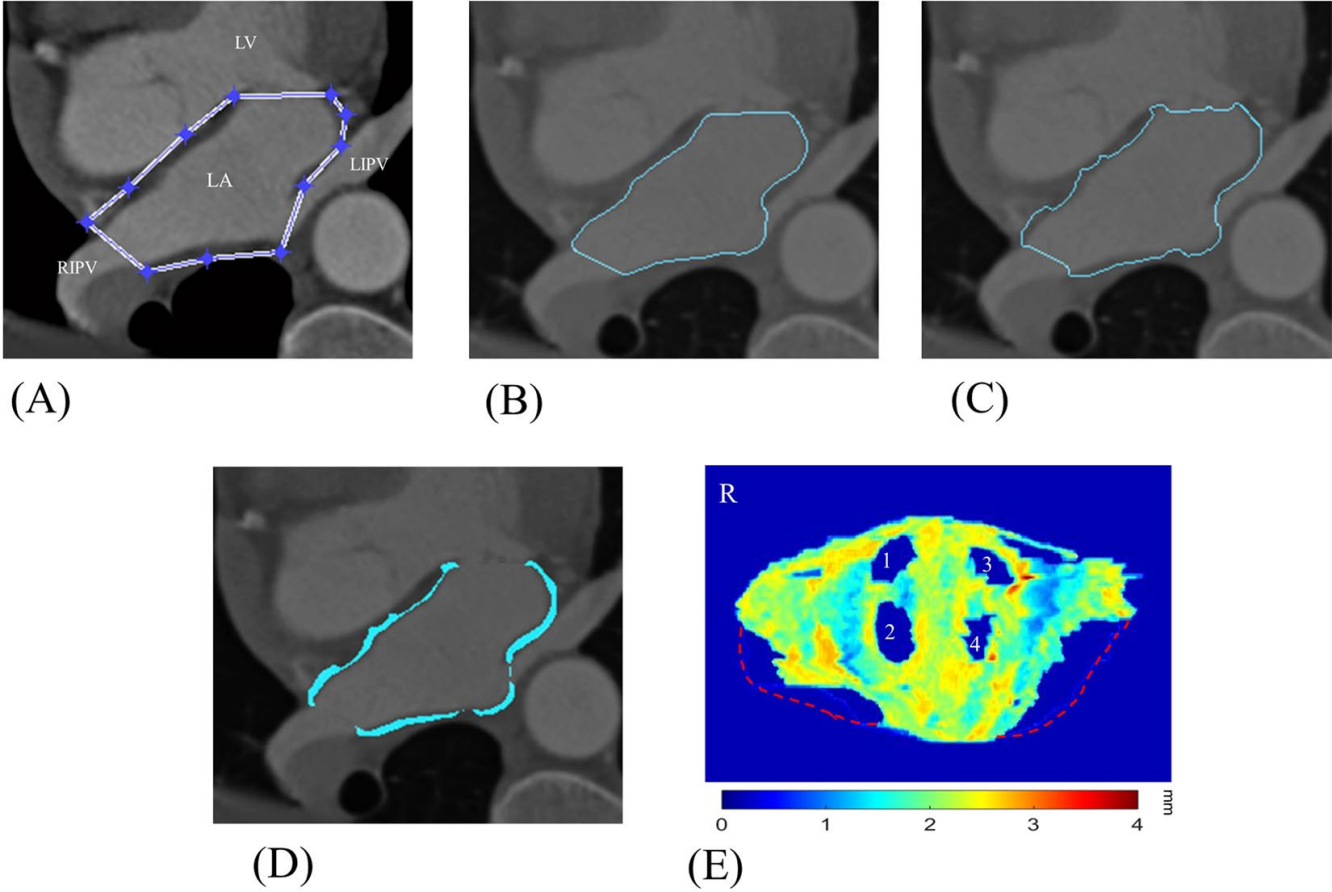
Procedures for processing and analysing a set of clinical CT images using the LA wall-mapping application. (**A**) LA delineation; (**B**) inner-boundary segmentation; (**C**) outer-boundary segmentation; (**D**) wall thickness calculation; and (**E**) 2D wall map generation. The colour bar shows the wall thickness. 1: right superior pulmonary vein (RSPV); 2 right inferior pulmonary vein (RIPV); 3 left superior pulmonary vein (LSPV); and 4: left inferior pulmonary vein (LIPV). The red dashed line indicates the annulus of mitral valve (MV).

**Figure 10:**
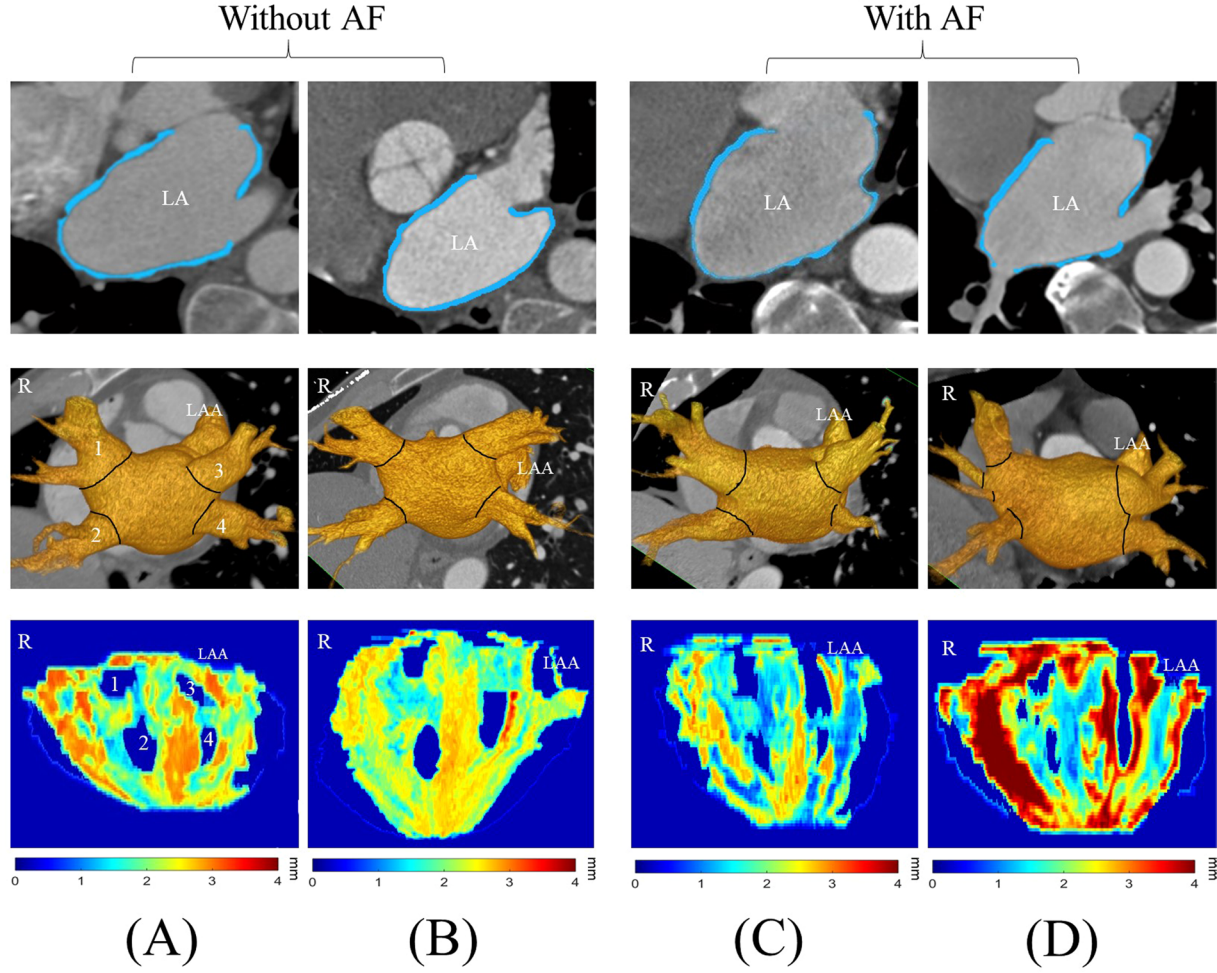
Sampleclinical results. The columns (**A**) and (**B**) show the results of the two patients without AF, and the columns (**C**) and (**D**) show the results of the two AF patients.The axial CT images, the LA surface-rendered model, and the wall map results are shown in the top, middle, and bottom row, respectively. The LA wall is marked in blue, and the black solid line represents the intersectionbetween the LA and PV. Labels 1-4 indicatethe ostium of pulmonary veins.The colour bar shows the wall thickness.

**Figure 11:**
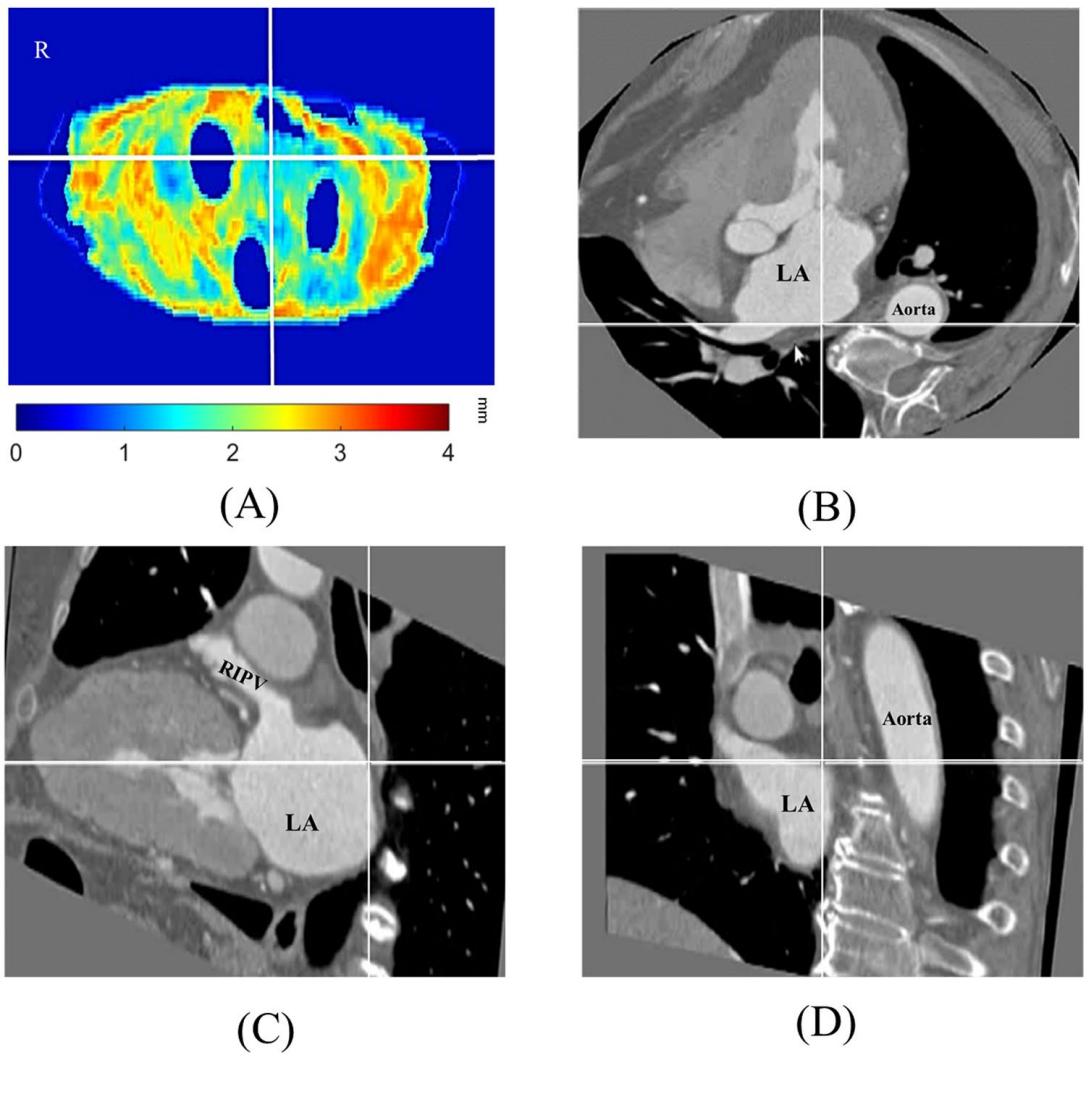
A specific position on the (**A**) LA wall map can be simultaneously localised on the corresponding cardiac CT images in (**B**) a 4-chamber view, (**C**) a 2-chamber view, and (**D**) a short-axis view. RIPV: right inferior pulmonary vein.

The comparisons of LA wall thickness measures between the pig heart specimens (Fig. 7A) and corresponding LA expanded wall map (Fig. 7B,C) by our mapping application were displayed in Fig. 7. The mean LA wall thickness of specimens was 1.99 ± 1.41 mm and 1.86 ± 1.30 mm for CT-based measures, which showed no significant differences between specimen and CT-based analysis (*p* = 0.11). There was good correlation between measures of pig heart specimens and CT-based mapping (Fig. 8, Pearson correlation *r* = 0.96, *p* < 0.001). Figure 9 showed the image analysis results of the clinical cardiac CT images from step 1 to 5. Of the eight patients in this study, six were men. The mean age was 57 (49–74) years, and the mean heart rate was 76 ± 11.2 bpm. Figure 10 shows the LA wall maps for four patients, and each LA wall map was calculated using the average wall thickness and heterogeneity per global LA area. For patients without AF, the average LA wall thickness were 2.03 mm (Fig. 10A) and 1.89 mm (Fig. 10B). For patients with AF, the average LA wall thickness were 1.62 mm (Fig. 10C) and 2.45 mm (Fig. 10D). The degree of the heterogeneity was 0.55 and 0.57 for patients without AF patients (Fig. 10A and B), and was 0.71 and 1.07 for patients with AF (Fig. 10C and D). Our findings demonstrate that the LA wall-mapping application can successfully visualise the LA wall thickness. Figure 11 shows the simultaneously localisation of the wall thickness map with cardiac CT images in the 4-chamber view, 2-chamber view, and short axial view. The processing time for the application of one set of CT images was approximately 30 minutes. The intra-observer and inter-observer reproducibility for eight sets of cardiac CT images were ICC > 0.90 (95% CI 0.82–0.99) and ICC > 0.8 (95% CI 0.71–0.99), respectively (Table 3).

**Table 3:** Intra-observer and inter-observer reproducibility of LA wall-mapping application from the clinical patient study.

	LA wall map	Intra-observer ICC	95% CI	Inter-observer ICC	95% CI
Chamber volume	99.17 ± 16.97 cm^3^	0.99	0.98–0.99	0.99	0.96–0.99
Wall volume	13.82 ± 3.47 cm^3^	0.98	0.94–0.99	0.94	0.90–0.99
Wall thickness	1.89 ± 0.2 mm	0.99	0.97–0.99	0.94	0.82–0.99
RSPV	26.2 ± 10.8 mm^2^	0.94	0.83–0.96	0.81	0.72–0.93
RIPV	30.6 ± 10.2 mm^2^	0.95	0.88–0.97	0.81	0.72–0.93
LSPV	19.5 ± 6.40 mm^2^	0.94	0.82–0.96	0.80	0.71–0.92
LIPV	16.4 ± 8.20 mm^2^	0.95	0.87–0.97	0.81	0.72–0.92

RSPV: right superior pulmonary vein; RIPV: right inferior pulmonary vein; LSPV: left superior pulmonary vein; LIPV: left inferior pulmonary vein.

## Discussion

Herein, we developed an LA wall-mapping application with the capabilities of visualisation and quantification of different LA structural parameters, by automatically processing and analysing the global LA wall thickness and displaying it on a coplanar plane. This application was validated using a self-designed LA phantom, six sets of pig cardiac CT images, specimens and eight sets of clinical cardiac CT images. The results demonstrated that the expanded map generated using the LA wall-mapping application had a high accuracy, and high intra-observer and inter-observer reproducibility.

Existing literature showed that the LA wall structural remodelling played an important role in AF. Regional, transmural characteristics of LA may form an anatomic re-entrant driver (RD) or “rotor” for more precise anatomic targetingin ablation^10,11^. In an autopsy study, Platonov *et al*. demonstrated that the LA posterior wall is generally thinner in patients with history of AF^4^. Nakamura *et al*. showed that the LA wall thickness in CT images might be a valuable predictor of the transition from paroxysmal atrial fibrillation (PAF) to chronic atrial fibrillation (CAF)^16^. Studies showed that except for the complexity of LA endocardial geometry, several features of LA transmural structures, as well as myoarchitecture, are challenging during LA ablation in patients with AF^1,2^.

Recently, a CT study showed the measurement results of 12 distinct LA locations in AF patients^7^, indicating regional non-uniform wall thickness in the LA wall in AF subjects. These studies demonstrated the importance of LA wall architecture in treating AF patients. On the other hand, MRI with late gadolinium enhancement (LGE) has also been used in the objective quantification of LA fibrotic tissue. Several studies explored the feasibility of LGE-MRI in identifying both pre-existing and post-ablation LA wall fibrosis^30,31^. It can not only identify the radiofrequency lesions in the myocardium, but may also improve the accuracy for identifying anatomic gaps in post-ablation cases^32^. Compared to CT, the advantage of LGE-MRI in LA wall assessment is better tissue characterization and no radiation exposure. Despite numerous advantages, LGE-MRI technique of LA wall has only been performed in a few experienced centres and not widely feasible or adopted in daily clinical practice due to its relatively highly technically dependency and poor reproducibility among groups^33,34^.In addition, MRI technique provides reconstructed images in sub-minimeter spatial resolution for analysis but the original transverse voxel size is about 1.25 × 1.25 × 2.5 mm. Advances in the 2nd generation dual source CT as we used in this study has currently allowed 0.33 mm isotropic spatial resolution, which can be superior to the resolution (0.5–0.6 mm) of traditional sixty-four slice MDCT^35^ or MRI. This improvement in spatial resolution further enables better visualization of smaller and distal coronary artery branches as well as the sub-minimeter thickness of left atrial wall.

In the present study, we proposed a novel LA wall-mapping application for the detailed assessment of global LA wall architecture, which may add additional clinical diagnostic value for the treatment of AF. It generally shows high accuracy, and high intra-observer and inter-observer reproducibility. However, as shown in Fig. 5, a few measurement errors were noted at the roof of the LA wall map for all three LA_measurement_ phantoms. This error is primarily attributed by the partial volume effect (PVE) in the CT images^29^. As shown in Fig. 6, PVE is more pronounced in regions with a large curvature. Once the roof wall thickness is increased because of the PVE, the chamber volume and height decrease, as shown in Table 1.

The PVE can be reduced by improving the spatial resolution of the CT image, which relies on the CT hardware improvement. Besides the roof, regions close to the junction of the PV and LA also possess high curvature in the clinical setting. The LA delineation of the LA wall-mapping application can separate the LA and PVs. Therefore, the analysis of clinical CT images would be less affected by the PVE. On the other hand, prior literature presents several methods of partial volume correction (PVC) based on image segmentation, such as fuzzy *c*-mean algorithm, expectation maximization algorithm, or generalized segmentation-based partial volume correction algorithm^36^. Those methods can calculate the fraction of each partial volume voxel from their reference values. In this regard, a better algorithm for PVC may be help in further studies. In our current work, we showed our CT-derived map application correlated well with real specimen on LA wall thickness measures from pig hearts.

While histological findings of non-uniform transmural myofiber distribution from diseased LA tissue had been well documented^37,38^, more objective and quantitative data on macroscopic LA surface morphology assessing local geometric complexity and heterogeneity have not been available. In addition, the link between altered atrial myofiber orientation and local geometry (e.g. thickness) has recently been proved to provide insights into pathophysiological basis of AF (e.g. as anatomic re-entry substrate) and serve as useful therapeutic targets^16,23,24^. Beyond LA volumes as commonly used metrics of LA remodeling or any functional measures by echocardiography or MRI^38^, indeed, image-based model may further improve our understandings of regional atrial muscular architecture on electrical properties and performance in various cardiac disorders.

Though a common pattern of general LA architecture has been noticed on the subendocardial surface of LA^39^, it has also been proposed that atrial remodeling or stretch from a variety of cardiac disorders (such as hypertension or heart failure) and aging may induce LA heterogeneous remodeling^40–42^, cellular fibrosis, and electrical conduction alterations^16^. Therefore, whether such atrial myofiber alterations or regional remodeling may serve as useful clinical prognosticators for any other form of atrial arrhythmias remained to be determined. As currently recommended by cardiac arrhythmia society, visualization of LA morphology from endocardial surface therefore provides an opportunity to clarify the concept that whether there might exist any associations between more early atrial architectural remodeling and the development of certain diseases, for example, AF or heart failure^43–45^.

These concepts can be in part illustrated from our four clinical patients (Fig. 10). We show that the distribution of the LA wall thickness may be affected by underlying cardiac pathology. The degrees of the inhomogeneity of the LA wall thickness were 0.55 and 0.57 for patients without AF patients and were 0.71 and 1.07 for patients with AF. Based on this, we demonstrated that the LA wall thickness tend to be more heterogeneous in the AF patients compared to the non-AF patients, which may be due to the myoarchitecture remodelling from elevated wall stress in AF^3^.

In conclusion, the LA wall-mapping application allows assessment and quantification of the LA wall using cardiac CT images. It showed good accuracy, and high intra-observer and inter-observer reproducibility. It may aid the LA ablation procedure when implemented in the electrophysiological mapping system.
